# The Reoperation, Readmission, and Complication Rates at 30 Days Following Lumbar Decompression for Cauda Equina Syndrome

**DOI:** 10.7759/cureus.49059

**Published:** 2023-11-19

**Authors:** Ryan Filler, Rusheel Nayak, Jacob Razzouk, Omar Ramos, Damien Cannon, Zachary Brandt, Savyasachi C Thakkar, Philip Parel, Anthony Chiu, Wayne Cheng, Olumide Danisa

**Affiliations:** 1 Department of Orthopaedic Surgery, Loma Linda University Medical Center, Loma Linda, USA; 2 Department of Orthopaedic Surgery, School of Medicine, Loma Linda University, Loma Linda, USA; 3 Spine Surgery, Twin Cities Spine Center, Minneapolis, USA; 4 School of Medicine, Loma Linda University, Loma Linda, USA; 5 Orthopaedic Surgery, Johns Hopkins Health System, Baltimore, USA; 6 Department of Orthopaedic Surgery, George Washington University School of Medicine and Health Sciences, Washington D.C., USA; 7 Department of Orthopaedics, University of Maryland, Baltimore, USA; 8 Division of Orthopaedic Surgery, Jerry L. Pettis VA Medical Center, Loma Linda, USA

**Keywords:** reoperation, readmission, pearldiver, lumbar decompression, cauda equina syndrome (ces)

## Abstract

Background and objective

Cauda equina syndrome (CES) is considered a surgical emergency, and its primary treatment involves decompression of the nerve roots, typically in the form of discectomy or laminectomy. The primary aim of this study was to determine the complication, reoperation, and readmission rates within 30 days of surgical treatment of CES secondary to disc herniation by using the PearlDiver database (PearlDiver Technologies, Colorado Springs, CO). The secondary aim was to assess preoperative risk factors for a higher likelihood of complication occurrence within 30 days of surgery for CES.

Methods

A total of 524 patients who had undergone lumbar discectomy or laminectomy for CES were identified. The outcome measures were 30-day reoperation rate for revision decompression or lumbar fusion, and 30-day readmissions related to surgery. The patient data collected included medical history and surgical data including the number of levels of discectomy and laminectomy.

Results

Based on our findings, intraoperative dural tears, valvular heart disease, and fluid and electrolyte abnormalities were significant risk factors for readmission to the hospital within 30 days following surgery for CES. The most common postoperative complications were as follows: visits to the emergency department (63 patients, 12%), surgical site infection (21 patients, 4%), urinary tract infection (14 patients, 3%), and postoperative anemia (11 patients, 2%).

Conclusions

In the 30-day period following lumbar decompression for cauda equina syndrome, our findings demonstrated an 8% reoperation rate and 17% readmission rate. Although CES is considered an indication for urgent surgery, gaining awareness about reoperation, readmission, and complication rates in the immediate postoperative period may help calibrate expectations and inform medical decision-making.

## Introduction

Cauda equina syndrome (CES) is a neurologic condition resulting from the compression of nerve roots of the cauda equina [1]. The reported incidence of CES is approximately one or two per 100,000, making it a relatively rare diagnosis [2,3]. Patients commonly present with symptoms including low back pain, radiculopathy of one or both lower extremities, saddle anesthesia, motor weakness of the lower extremities, and dysfunction of the bladder and, occasionally, bowel. It is most commonly caused by a herniated lumbar intervertebral disc [2]. Other causes of CES include traumatic injuries with bony compression or hematoma. Malignancy, such as metastatic disease or infection with epidural hematoma, can also cause CES. Multiple studies have reported poor long-term outcomes in patients with CES due to the high incidence of urinary, defecatory, sexual, and motor dysfunction [4,5]. Delay in diagnosing and treating CES also poses a significant risk for malpractice litigation [6-7]. Due to these and other factors, CES can lead to significant medical and social burdens as well as high healthcare costs [8].

The recommended treatment for CES involves surgical exploration and decompression of any lesions [9]. In CES secondary to disc herniation, surgical treatment can range from a simple microdiscectomy to a wide laminectomy with inspection of the thecal sac and nerve roots. Traditionally, surgical intervention is performed promptly, preferably within 24 hours [9]. Ahn et al. have reported improvement in neurologic outcomes in patients treated within 48 hours compared to those treated more than 48 hours after the onset of symptoms, but there was no difference in those who were treated between 24 and 48 hours [10]. Other studies have reported similar findings and hence concluded that these patients should be treated urgently within 48 hours [8,11,12].

The PearlDiver database (PearlDiver Technologies, Colorado Springs, CO) is a de-identified, commercially available, Humana-approved database with medical records of over 25 million patients from 2007 through 2017. As CES and associated complications are rare, the PearlDiver Patient Record Database may provide insights into significant trends and data related to CES that may be difficult to obtain otherwise. The primary aim of this study was to determine the complication, reoperation, and readmission rates within 30 days of undergoing surgical treatment for CES by using the PearlDiver database. The secondary aim was to assess preoperative risk factors associated with a higher likelihood of complications within 30 days of surgery for CES.

## Materials and methods

After obtaining institutional review board (IRB) approval (#5220054), a retrospective cohort study of patients undergoing surgical treatment of CES was conducted using the PearlDiver Patient Record Database. All patients undergoing lumbar discectomy and/or laminectomy based on a CES diagnosis were identified by using Current Procedural Terminology (CPT) codes 63030-63035 for primary discectomy, and code 63047 for lumbar laminectomy. The presence of cauda equina was determined based on the International Classification of Disease, Ninth Revision (ICD-9), and International Classification of Disease, Tenth Revision (ICD-10) diagnosis codes 344.60 and 344.61, and G83.4, respectively. The exclusion criteria were as follows: patients with a preoperative diagnosis of infection, spinal fracture, or neoplasm, or patients undergoing simultaneous lumbar fusion, spinal osteotomy, or vertebral column resection for correction of deformity.

The primary outcome measures analyzed were the 30-day reoperation rate for revision decompression or lumbar fusion, and 30-day readmissions related to surgery. Readmissions related to surgery were determined based on the presence of new or recurrent pain, neurological symptoms, or surgical site infection as indicated by recorded ICD-9 or ICD-10 codes. The demographic and surgical data collected included patient age, sex, BMI, number of levels of discectomy and laminectomy, revision discectomy, or dural tear during initial surgery. The data relating to past medical history, such as history of coronary artery disease, congestive heart failure, hyperlipidemia, arrhythmia, valvular disease, pulmonary circulatory disease, pulmonary disease, peripheral vascular disease, hypertension, paralysis, neurologic deficit, diabetes mellitus, hypothyroidism, chronic kidney disease, peptic ulcer disease, lymphoma, metastatic cancer, cancer without metastasis, rheumatoid arthritis, coagulopathy, fluid or electrolyte disorder, blood loss anemia, iron deficiency anemia, drug abuse, psychiatric disorder, depression, smoking, obesity, and alcohol abuse, were also collected. We specifically studied depression as a risk factor separate from psychiatric disorder, because depression, on its own, has been found to have significant negative effects on patient outcomes [13-19].

All statistical analyses were performed using R (v 3.6.3) statistical analysis software. All statistical analyses were two-tailed with the alpha set to 0.05 for denoting statistical significance. Descriptive statistics were presented as mean ± standard deviation (SD) for all continuous data, and frequency counts and percentages for all categorical data. Statistical differences between groups with respect to continuous variables were assessed using independent sample t-tests. Statistical differences between groups with respect to categorical variables were assessed using Pearson's chi-squared test with post hoc Bonferroni correction applied for explanatory variables containing three or more groups. Two forms of power analyses were performed. Nondirectional partial correlation power analysis was conducted with a sample size of 90, specified parameters of 0.05 for alpha, a null value of zero, a partial correlation parameter of 0.300, and an assumed number of four variables to be partialled out. Power analysis determined an achieved power of 97.2%. The chi-square goodness-of-fit power analysis with an effect size of 0.5, a sample size of 90, and 6 digits demonstrated a test power of 0.94 and a critical value of 16.92.

## Results

Our analysis identified a total of 524 patients who underwent lumbar discectomy or laminectomy for cauda equina syndrome. Of the 524 patients, 42 (8%) underwent reoperation within 30 days and 91 (17%) were readmitted to the hospital for reasons related to surgery within the first 30 days. None of the preoperative or intraoperative risk factors evaluated in our study were found to be significantly associated with reoperation within 30 days postoperatively (Table 1). However, intraoperative dural tears, valvular heart disease, and fluid and electrolyte abnormalities were found to be significant risk factors for readmission to the hospital within the first 30 days following surgery (Table 2). Of the 91 patients readmitted within the first 30 days, 18.68% had intraoperative dural tears compared to only 6.47% of control patients not readmitted (p<0.001); 19.78% had valvular heart disease compared to only 8.08% in the control group (p=0.002); and 34.07% had a fluid and electrolyte disorder compared to 22.86% in the control group (p=0.034). No other risk factors evaluated in our study were found to be significantly associated with readmission within 30 days.

**Table 1:** Comparison of 30-Day Reoperation Demographics and Comorbidities (Chi-Square Test)

Risk Factor (n=524)	Reoperation Group (%)	Control Group (%)	P-value
Male Sex	47.62%	48.76%	1
Far Lateral Discectomy	0.00%	6.85%	0.1554
Dural Tear	14.29%	8.09%	0.277
Coronary Artery Disease	23.81%	23.03%	1
Hyperlipidemia	64.29%	65.15%	1
Congestive Heart Failure	4.76%	7.26%	0.7699
Arrhythmia	19.05%	24.27%	0.5663
Valvular Disease	9.52%	10.17%	1
Pulmonary Circulatory Disorder	4.76%	3.73%	1
Peripheral Vascular Disease	11.90%	15.35%	0.7083
Hypertension (Uncomplicated)	28.57%	45.44%	0.0513
Paralysis	30.95%	24.07%	0.4198
Neurological Disorder	42.86%	51.04%	0.392
Pulmonary Disease	16.67%	24.69%	0.3278
Diabetes Mellitus	28.57%	24.27%	0.6648
Hypothyroidism	14.29%	15.98%	0.9464
Chronic Kidney Disease	11.90%	10.17%	0.9276
Peptic Ulcer Disease	4.76%	1.87%	0.4877
Lymphoma	0.00%	0.83%	1
Metastatic Cancer	2.38%	3.11%	1
Cancer Without Metastasis	7.14%	11.20%	0.5808
Rheumatoid Arthritis/Collagen Disorder	11.90%	14.52%	0.8142
Coagulopathy	7.14%	4.36%	0.6574
Fluid and Electrolyte Disorder	30.95%	24.27%	0.4384
Blood Loss Anemia	4.76%	2.90%	0.8388
Deficiency Anemia	16.67%	11.62%	0.4731
Drug Abuse	2.38%	10.37%	0.1601
Psychoses	9.52%	3.73%	0.1636
Depression	38.10%	34.02%	0.7163
Smoking	21.43%	22.20%	1
Obesity	11.90%	17.84%	0.4461
Alcohol Abuse	0.00%	1.04%	1

**Table 2:** Comparison of 30-Day Readmission Demographics and Comorbidities (Chi-Square Test)

Risk Factor Description (n=524)	Readmission Group (%)	Control Group (%)	P-value
Male Sex	40.66%	50.35%	0.1175
Far Lateral Discectomy	5.49%	6.47%	0.9127
Dural Tear	18.68%	6.47%	0.0004
Coronary Artery Disease	26.37%	22.40%	0.4962
Hyperlipidemia	62.64%	65.59%	0.6775
Congestive Heart Failure	6.59%	7.16%	1
Arrhythmia	25.27%	23.56%	0.8303
Valvular Disease	19.78%	8.08%	0.0015
Pulmonary Circulatory Disorder	3.30%	3.93%	1
Peripheral Vascular Disease	17.58%	14.55%	0.5661
Hypertension (Uncomplicated)	42.86%	44.34%	0.8862
Paralysis	28.57%	23.79%	0.407
Neurological Disorder	49.45%	50.58%	0.9362
Pulmonary Disease	26.37%	23.56%	0.6623
Diabetes Mellitus	28.57%	23.79%	0.407
Hypothyroidism	19.78%	15.01%	0.3297
Chronic Kidney Disease	15.38%	9.24%	0.1179
Peptic Ulcer Disease	2.20%	2.08%	1
Lymphoma	0.00%	0.92%	0.7965
Metastatic Cancer	3.30%	3.00%	1
Cancer Without Metastasis	12.09%	10.62%	0.8238
Rheumatoid Arthritis/Collagen Disorder	14.29%	14.32%	1
Coagulopathy	7.69%	3.93%	0.1983
Fluid and Electrolyte Disorder	34.07%	22.86%	0.0344
Blood Loss Anemia	4.40%	2.77%	0.6287
Deficiency Anemia	17.58%	10.85%	0.106
Drug Abuse	10.99%	9.47%	0.8024
Psychoses	6.59%	3.70%	0.3342
Depression	41.76%	32.79%	0.1297
Smoking	21.98%	22.17%	1
Obesity	23.08%	16.17%	0.1528
Alcohol Abuse	1.10%	0.92%	1

Apart from reoperation and readmission, other postoperative complications encountered in the first 30 days after surgery for CES were as follows: visits to the emergency department (63 patients, 12%), surgical site infection (21 patients, 4%), urinary tract infection (14 patients, 3%), and postoperative anemia (11 patients, 2%) (Table 3).

**Table 3:** Postoperative Complications CES: cauda equina syndrome; ED: emergency department; UTI: urinary tract infection; Afib: atrial fibrillation; DVT: deep vein thrombosis

Complication	Number of Patients	%
Patients Receiving Surgery For CES	524	100%
Revision Within 30 Days	42	8%
No Revision (30 Days)	482	92%
Readmission Within 30 Days	91	17%
No Readmission (30 Days)	433	83%
ED Visit	63	12%
Surgical Site Infection	21	4%
UTI	14	3%
Postoperative Anemia	11	2%
Renal Failure	0	0%
Arrhythmia with Afib	0	0%
Arrhythmia without Afib	0	0%
Blood Transfusion	0	0%
Death	0	0%
DVT	0	0%
Heart Failure	0	0%
Pulmonary Embolism	0	0%
Pneumonia	0	0%
Respiratory Complication	0	0%
Sepsis	0	0%
Cerebrovascular Accident	0	0%
Bleeding Complication	0	0%
Myocardial Infarction	0	0%
Transient Mental Disorder	0	0%
Intubation	0	0%
Ileus	0	0%

No clear relationship was found between age or BMI and rates of revision or readmission within 30 days after surgery for CES. There was a slight increase in revision and readmission rates for patients with BMI over 40 though this did not reach statistical significance (Tables 4, 5).

**Table 4:** Age as a Risk Factor for 30-Day Reoperation or Readmission (T-Test)

	Risk Factor	N (Total)	N (Reoperation/Readmission)	Mean Age (Reoperation/Readmission), Years	N (Control)	Mean Age (Control), Years	P-value
Reoperation	Age	524	42	51.7619	482	53.66598	0.4204
Readmission	Age	524	91	53.69231	433	53.5127	0.9155

**Table 5:** BMI as a Risk Factor for 30-Day Reoperation or Readmission BMI: body mass index

BMI, KG/M^2^	No Revision	Revision Within 30 Days	%
<20	0	0	N/A
20-25	24	2	7.70%
25-30	59	5	7.80%
30-35	103	5	4.60%
35-40	78	6	7.10%
>40	100	12	10.70%
X-squared: 8.1101, p-value: 0.1503			
BMI	No Readmission	Readmission Within 30 Days	%
<20	0	0	N/A
20-25	22	4	15.40%
25-30	50	14	21.90%
30-35	85	23	21.30%
35-40	65	19	22.60%
>40	84	28	25.00%
X-squared: 3.8189, p-value: 0.5758			

## Discussion

This is a large database study analyzing short-term readmission, reoperation, and complication rates following lumbar decompression performed in the setting of CES. Reoperation and readmission are costly and burdensome for payers, hospitals, and patients alike. Both reoperation and readmission rates are being increasingly employed as performance metrics, with penalties often imposed for excessive readmission.

Reoperation

Our study found an 8% rate of 30-day reoperation for revision decompression or lumbar fusion in patients with CES who underwent discectomy or laminectomy. This is a significantly higher rate than what is reported in the literature for these same procedures when performed on an elective basis. Golinvaux et al. reported a 30-day reoperation rate of 2% in patients in the NSQIP database after elective discectomy, which was compared to a 4% one-year reoperation found in the SPORT trial, with more than 50% of these occurring due to recurrent disc herniation [14]. A study of patients undergoing surgery for lumbar stenosis found a 90-day reoperation rate of 4.7%, with 70% of the reoperations occurring within the first 30 days, with the risk factors for reoperation being development of SSI, sepsis, UTI, and increased length of stay [18]. Ambrossi et al. reported a 7% rate of revision surgery for patients with same-level recurrent disc herniation after single-level lumbar discectomy within 12 months [19]. A systematic review by McGirt et al. determined that mean incidence rates of recurrent disc herniation following limited discectomy and aggressive discectomy were 7% and 3.5%, respectively [20].

Readmission

Our study found a 17% readmission rate with dural tears, valvular heart disease, and fluid and electrolyte disorders demonstrating risk factors for readmission. This is a significantly higher readmission rate than what is reported in the literature for lumbar decompression performed for reasons other than CES. Reito et al. analyzed 130 patients undergoing urgent lumbar discectomies, excluding those with CES, and found a 30-day readmission rate of 6.9% [13]. However, studies with similar methodology have reported rates between 2.6% and 3.7% [18,21,22].

Durotomy has been previously found to pose a risk factor for readmission, which is in line with our findings [16]. ASA class, prolonged operative time, length of stay, and age over 80 years have also been associated with increased risk for readmission after lumbar decompression [20]. Ilyas et al. found a 90-day readmission rate of 7.2% after surgery for lumbar stenosis with significant risk factors including surgical site infection, acute kidney injury, urinary tract infection, and history of congestive heart failure [23]. An NSQIP database study of patients undergoing elective lumbar decompression calculated a readmission rate of 4.4% with anemia, dependent functional status, total operative time, and the American Society of Anesthesiologists Physical Status Class 4 as independent predictors of readmission [24]. Other analyses in the setting of elective lumbar decompression have shown 30-day readmission rates ranging from 2.3% to 7.8% [25,26].

The sharp contrast between the reoperation and readmission rates seen in our study compared to the above studies may be a reflection of the more severe pathology of CES [25]. Since no difference has been shown in outcomes between operating in the first 24 hours versus between 24 and 48 hours, it may be reasonable to briefly delay surgery for CES patients under specific circumstances where preoperative factors can be feasibly optimized [10,26,27]. Additionally, operating outside normal operating room hours (e.g., during nighttime) has been shown to be an independent risk factor for complications in spine surgery performed for CES [28,29,30]. Preoperative optimization may help equilibrate outcomes after lumbar decompression performed in the setting of CES closer to those of lumbar decompression performed electively.

Complications

The most common complication identified in our study was an ED visit within 30 days. Age, current smoking status, longer hospital length of stay, and history of renal failure have been previously associated with complications after lumbar decompression surgery in one single institution study [31]. Jain et al. found a 12.8% rate of 30-day ED visits, primarily for pain and cardiorespiratory complaints in patients who had undergone lumbar fusion [32].

BMI

Though BMI has been shown to be a risk factor for surgical site infection, which in turn, has been shown to be a risk factor for both reoperation and readmission, our analysis did not detect a direct association between the two. This is in line with previous work demonstrating that BMI does not affect complications or outcomes after lumbar decompression [32].

Limitations

This study has a few limitations. As the PearlDiver database consists of billing data, it is possible that misclassification or misrepresentation of complications may have occurred. In addition, since the dataset is an aggregation of nationwide Humana patient data, we were unable to view individual records to obtain further context and granularity of data into the specifics behind complication rates, readmission rates, and reoperation rates. As such, we are unable to discern the exact etiology of the reasons necessitating reoperation, such as recurrent disc herniation, washout of infection, evacuation of a hematoma, or CSF leak. Consequently, the nature of our study design may be limited with regard to the completeness of its documentation as afforded by the PearlDiver database. Furthermore, while the database reflects nationwide data pertaining to a large, diverse population, it fundamentally represents a subpopulation among those with health insurance, which may differ from the overall United States population.

## Conclusions

Based on our findings, in the 30-day period following lumbar decompression for CES, there was an 8% reoperation rate and a 17% readmission rate. Readmissions were associated with intraoperative dural tears, valvular heart disease, and fluid and electrolyte disorders. However, reoperation within the 30-day postoperative window was not associated with any of the patient factors assessed in this study. Although CES is considered an indication for urgent surgery, being equipped with a thorough understanding of reoperation, readmission, and complication rates in the immediate postoperative period may help to calibrate expectations and inform medical decision-making.
